# Accuracy of Frozen Section Biopsy in the Diagnosis of Endometrial Cancer: A Systematic Review and Meta-Analysis

**DOI:** 10.3390/cancers16061200

**Published:** 2024-03-19

**Authors:** Stergios Kopatsaris, Aikaterini Apostolopoulou, Ioannis Tsakiridis, Antigoni Tranidou, Fotios Zachomitros, Evangelos Papanikolaou, Alexandros Daponte, Ioannis Kalogiannidis, Themistoklis Dagklis

**Affiliations:** 1 Third Department of Obstetrics and Gynecology, School of Medicine, Faculty of Health Sciences, Aristotle University of Thessaloniki, 54124 Thessaloniki, Greece; stkopats@auth.gr (S.K.); atranidou@auth.gr (A.T.); zfotios@auth.gr (F.Z.); evpapanikolaou@auth.gr (E.P.); ikalogia@auth.gr (I.K.); dagklis@auth.gr (T.D.); 2 Laboratory of Hygiene, Social & Preventive Medicine and Medical Statistics, School of Medicine, Faculty of Health Sciences, Aristotle University of Thessaloniki, 54124 Thessaloniki, Greece; aapostod@auth.gr; 3 Department of Obstetrics and Gynecology, School of Medicine, Faculty of Health Sciences, University of Thessaly, 41110 Larisa, Greece; daponte@med.uth.gr

**Keywords:** endometrial lesions, endometrial cancer, atypical hyperplasia, frozen section, diagnostic accuracy, meta-analysis

## Abstract

**Simple Summary:**

Endometrial cancer continues to be among the most prevalent cancers affecting the female reproductive system. The timely and precise diagnosis of endometrial cancer is crucial for the survival of individuals affected by it. The aim of the present systematic review and meta-analysis was to consolidate and assess the findings concerning the diagnostic precision of frozen section analysis of endometrial tissues for diagnosing endometrial cancer and atypical hyperplasia. The method has demonstrated high reliability in diagnosing endometrial cancer and even greater accuracy for atypical hyperplasia. This could significantly influence clinical practice, as a less invasive procedure such as frozen section biopsy could benefit a substantial number of women.

**Abstract:**

The early and accurate diagnosis of endometrial cancer is of paramount importance for the survival of these patients. The aim of this study was to systematically appraise the available data regarding the accuracy of frozen section biopsy in diagnosing endometrial cancer. A thorough literature search was performed in PubMed/Medline, Scopus and the Cochrane Central Register of Controlled Trials databases from inception up to January 2023, with the use of specific, relevant key terms. A quality evaluation for each study was performed with the QUADAS-2 tool, whereas a bivariate random-effect model was performed to generate a summary receiver-operated curve. Heterogeneity was evaluated with Cochrane Q and Higgins’ I2 statistics. Subgroup analyses were performed for studies focused on atypical hyperplasia and those focused on endometrial cancer. The search yielded 47 studies, involving 7790 patients with endometrial cancer. Among them, only 11 could be included in the quantitative analysis. QUADAS-2 evaluation resulted in rather high quality among the included studies. Quantitative synthesis resulted in a pooled sensitivity of 0.863 and pooled specificity of 0.916. The AUC was 0.948, the Q statistic was 10.488 (10 df, *p* = 0.399) and Higgins’ I^2^ (4.655%) reported no significant heterogeneity. Subgroup analyses based on the diagnosis revealed a pooled sensitivity 0.886, specificity 0.862 and AUC 0.934 for endometrial cancer versus a sensitivity of 0.816, specificity of 0.962 and AUC 0.939 for atypical hyperplasia. Frozen section appears as a valid and reliable diagnostic tool for endometrial cancer. Its reliability seems to be even higher for the diagnosis of atypical hyperplasia. Therefore, this method may be considered in clinical practice and in settings with appropriate resources.

## 1. Introduction

Endometrial cancer is one of the most common cancer types affecting women in both high- and low/middle-income countries [1]. The disease burden of endometrial cancer appears to be increasing as annual rates have increased from 0.58 to 0.89% between 1990 and 2017 worldwide [1]. The widely accepted gold standard treatment for endometrial cancer is a comprehensive procedure known as total hysterectomy combined with bilateral salpingo-oophorectomy. This surgery aims to remove the uterus along with the fallopian tubes and ovaries [2]. Various surgical approaches can be employed, including abdominal, laparoscopic, or robotic methods. It is noteworthy that minimally invasive techniques such as laparoscopic and robotic surgeries have been found to be equally effective as abdominal hysterectomy, offering patients potentially quicker recovery times and reduced postoperative discomfort [3]. In addition, lymphadenectomy, with pelvic/paraaortic lymph nodes removal, may be performed, according to the stage of the disease [2]. However, the extent and its therapeutic benefits on survival rates are still under investigation, especially in early cancer stages; some clinicians favor the removal of lymph nodes from all patients, whereas others choose to proceed with this procedure only in selected cases [4,5]. Sentinel lymph biopsy may be a safe alternative to systematic lymphadenectomy [6].

In most patients, tumor grade and histotype are determined preoperatively via endometrial curettage or biopsy. However, pathological examination during surgery demonstrates high sensitivity and specificity and may play a crucial role in surgical decisions [7,8]. In particular, relevant research has shown that frozen sections of endometrial tissues are highly predictive of the final diagnosis and the degree of surgical invasion required for each patient [9].

The aim of the present study was to synthesize and quantify findings regarding the diagnostic accuracy of endometrial tissues’ frozen section for the diagnosis of endometrial cancer.

## 2. Materials and Methods

This systematic review and meta-analysis was carried out in accordance with the Preferred Reporting Items for Systematic reviews and Meta-Analyses (PRISMA) guidelines [9]. The study protocol was registered with PROSPERO international prospective register of systematic reviews (protocol number: CRD42023389536).

### 2.1. Search Strategy

Two independent researchers (S.K. and A.A.) separately reviewed online databases, including PubMed/Medline, Scopus and the Cochrane Central Register of Controlled Trials (CENTRAL), from inception to 26 January 2023, searching for studies examining the diagnostic accuracy of frozen section for endometrial cancer. All references were inserted in a reference manager tool (Zotero) to identify and remove any duplicate studies. The initial search was performed by screening the title and abstract of each study, followed by a full text review by two independent researchers. Any disagreements were resolved by a third investigator (I.T.).

The literature search was performed using the following keywords: “frozen section, biopsy, endometrial cancer, endometrial malignancy, endometrial tumor”. The reference lists of each study included in this review were also carefully examined to identify potentially relevant papers that were not traced during the initial search.

With respect to the inclusion criteria, a study had to be published in English and to evaluate frozen section for the diagnosis of endometrial cancer compared to other diagnostic methods. A study was excluded when a different type of cancer was investigated, if the study aimed to evaluate the depth of invasion, or in cases where the provided data were insufficient. Moreover, a study that included both endometrial cancer and atypical hyperplasia, could be included in the review only in the case where it presented separate results regarding the two pathologies. Exclusion criteria related to the year of publication were not applied. Furthermore, the electronic registry of systematic reviews was examined to identify any previous meta-analyses on the topic.

### 2.2. Data Extraction

Data extraction was performed by using a standard predefined data form created in a datasheet file. Data collected included the first author, year of publication, journal, origin, as well as the basic characteristics of each study sample, data related to the frozen section and final biopsy procedure, the key findings of each study and any additional information necessary to assess the quality of the studies. In addition, the number of true positives, true negatives, false positives and false negatives was retrieved, and 2 × 2 tables were created for each study.

### 2.3. Quality Evaluation

A quality evaluation of each study was performed with the QUADAS-2 tool. QUADAS-2 is a well-known tool used for systematic reviews in order to assess risk of bias and applicability in primary research of diagnostic accuracy. QUADAS-2 consists of four main areas: sample selection, diagnostic criteria, reporting method and flow and timing. Each was assessed for risk of bias, with the first three also used to evaluate risk related to study implementation [10].

### 2.4. Quantitative Synthesis and Meta-Analysis

A bivariate random-effect meta-analysis was conducted according to the method described by Reitsma et al. [11]. This generated a summary receiver operating characteristic (SROC) with a calculated area under the curve (AUC) and a summary estimate of sensitivity and specificity, with confidence intervals (CI) creating a 95% confidence region ellipse on the SROC. A diagnostic odds ratio (DOR) was also calculated. The heterogeneity was evaluated with Cochrane Q and Higgins’ I^2^ statistics. Spearman’s correlation analysis between sensitivity and false positive rate was also calculated to consider threshold effect (r ≥ 0.6 generally indicates considerable threshold effect). A subgroup analysis based on the type of diagnosis (endometrial cancer or atypical hyperplasia) was also performed. All analyses were conducted in R using the mada package [12].

## 3. Results

### 3.1. Study Selection

**Figure 1:**
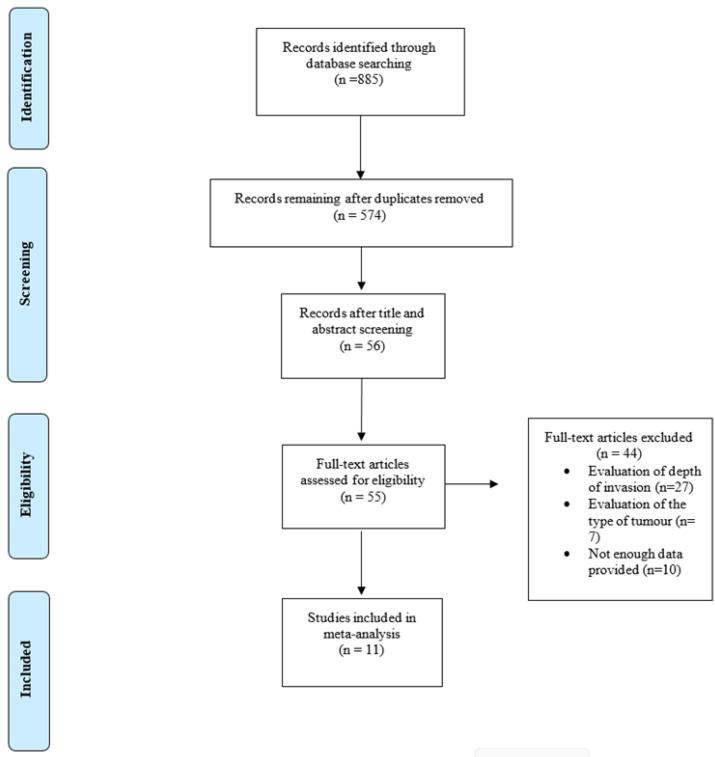
Flow chart of the included studies.

The initial search yielded 885 articles. After excluding duplicates, 574 articles remained. Subsequently, each title and abstract were screened, and 478 articles were excluded as they were either irrelevant to the topic or were published in other languages other than English. The 96 articles were retrieved as full-text and the eligibility criteria were applied by the researchers. The final step of the literature search yielded 47 studies conducted between 1993 and 2022 (the coefficient of agreement between the two reviewers was Cohen’s k: 0.911). Τhe study selection process is presented in Figure 1.

Most of the studies were retrospective cross-sectional studies that used data from medical records. In total, the 47 studies included 8353 patients; 41 of these studies included only patients with endometrial cancer (7790 cases), 7 of them encompassed 563 patients and included only cases of atypical hyperplasia, and 1 study reported separate results for the two diagnoses. Overall, only 11 studies were eligible for quantitative synthesis. Frozen section pathology was compared with the final pathology report. Regarding the surgical techniques, laparoscopic or abdominal total hysterectomy was performed in all cases. Frozen section was used to intra-operatively estimate the depth of myometrial invasion and decide subsequently whether to proceed to lymphadenectomy. The main characteristics of study groups are presented in detail in Table 1 and Table 2.

### 3.2. Quality Evaluation

As already mentioned, the evaluation of the studies was carried out by applying the QUADAS-2 tool. The results of the evaluation revealed that the overall quality of the included studies was rather high, as the only issues raised included the study design and the sampling procedure, which was not randomized. The results of the evaluation are presented in Supplementary Table S1.

### 3.3. Quantitative Analysis

**Figure 2:**
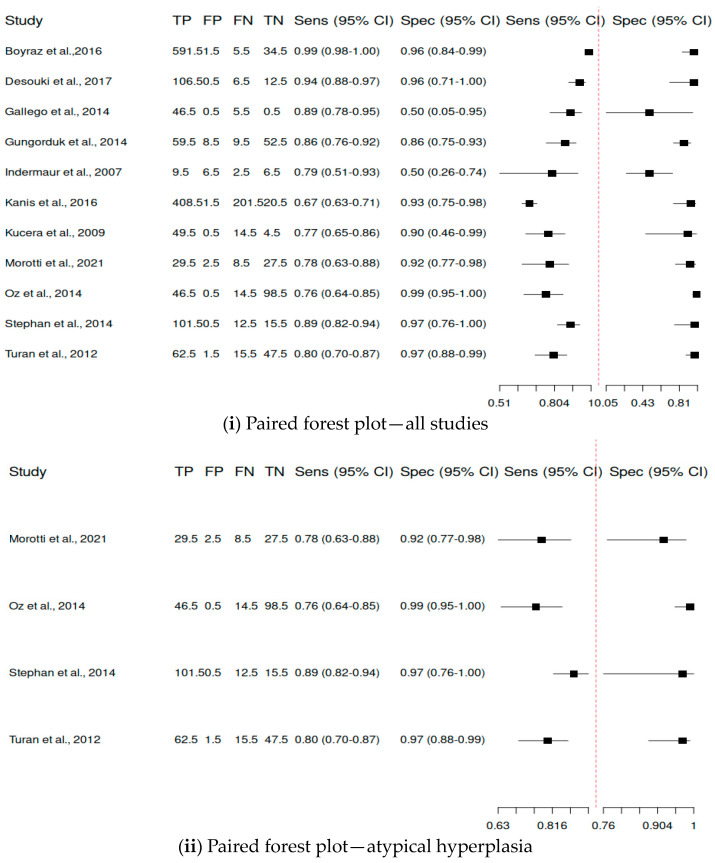

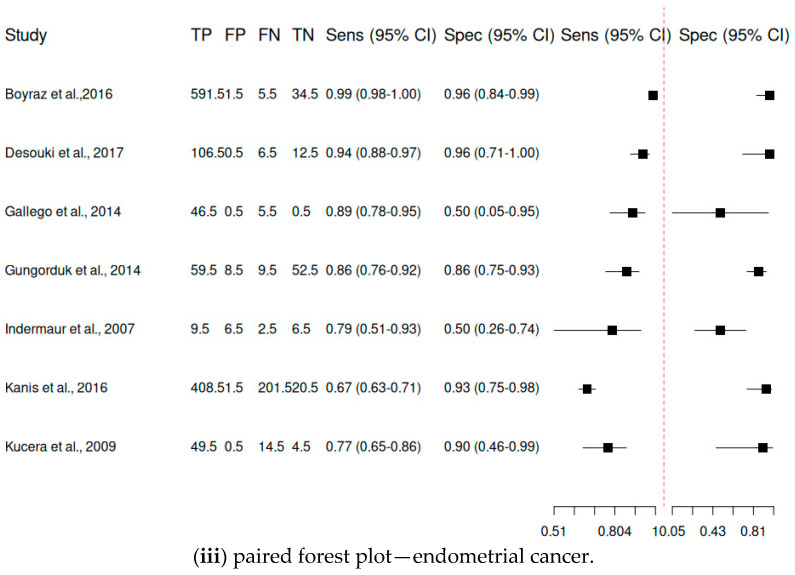
Sensitivity and specificity results for each study are presented [13,16,20,24,46,52,53,54,55,56,58].

**Figure 3:**
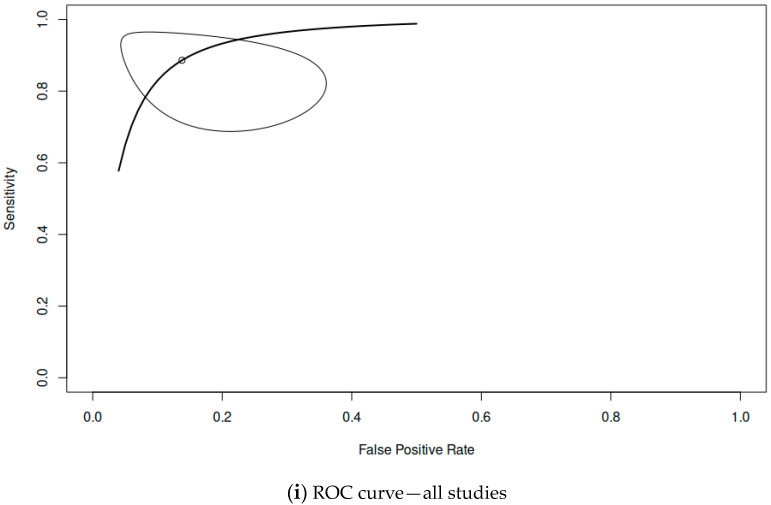

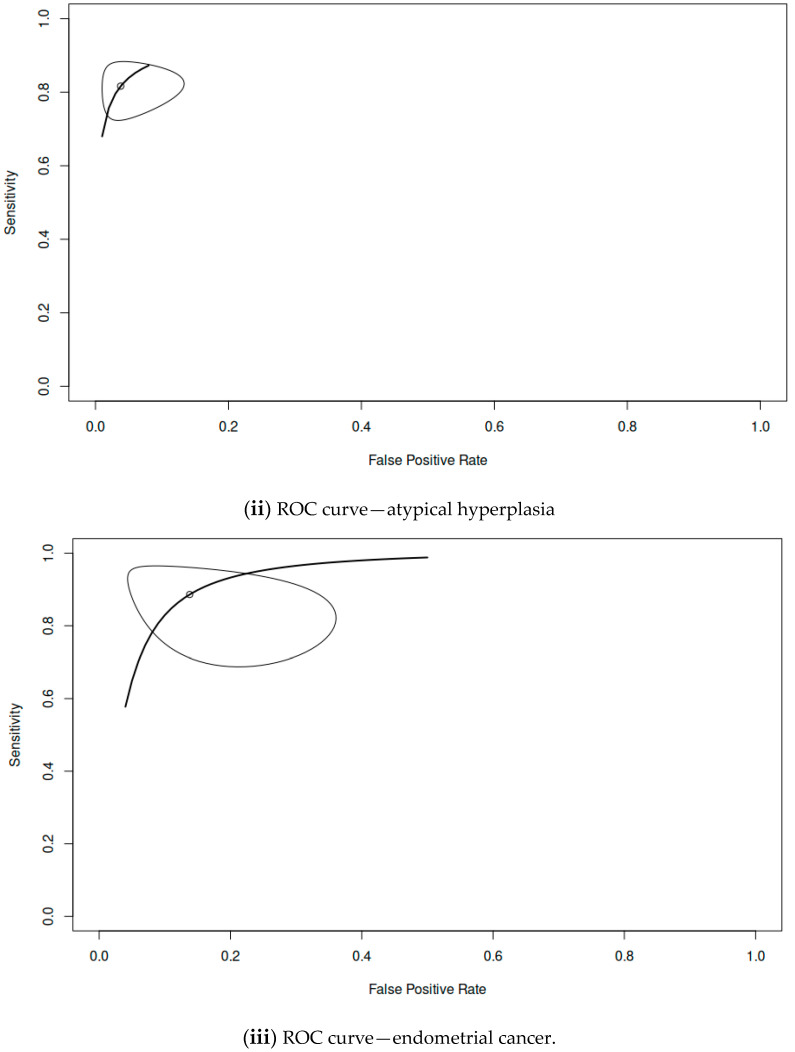
The ROC (receiver operating characteristic) curve is a graphical representation used to assess the performance of a classification model or a diagnostic test. It illustrates the trade-off between sensitivity (true positive rate) and specificity (true negative rate) across various thresholds. A perfect classifier would have an ROC curve that passes through the upper-left corner of the plot, indicating 100% sensitivity and 100% specificity.

**Figure 4:**
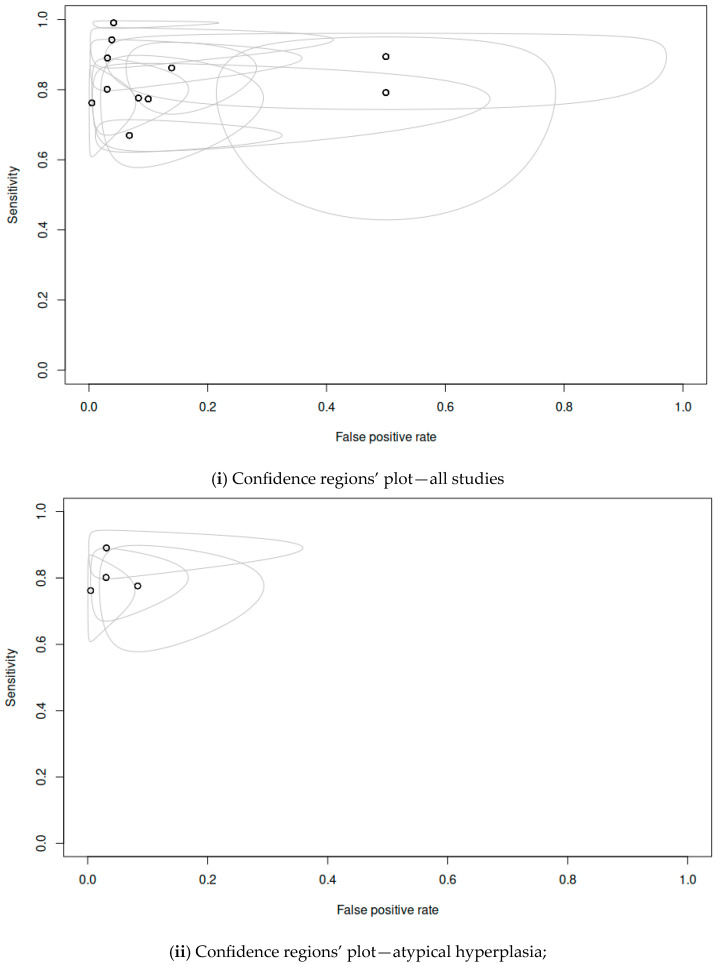

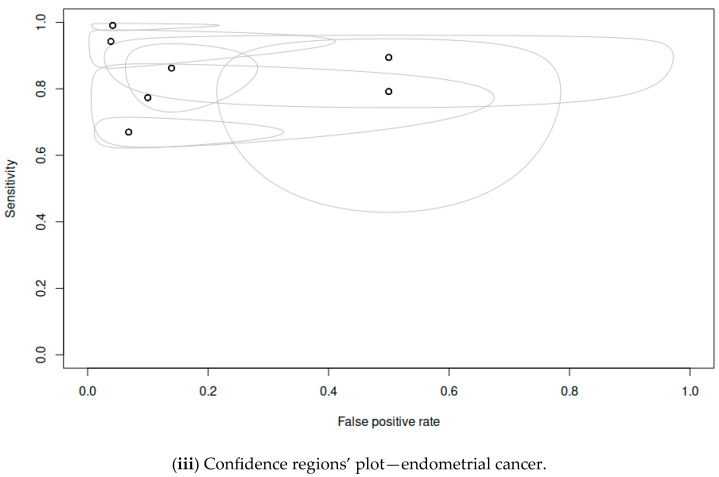
Plots with confidence regions for primary study estimates.

**Figure 5:**
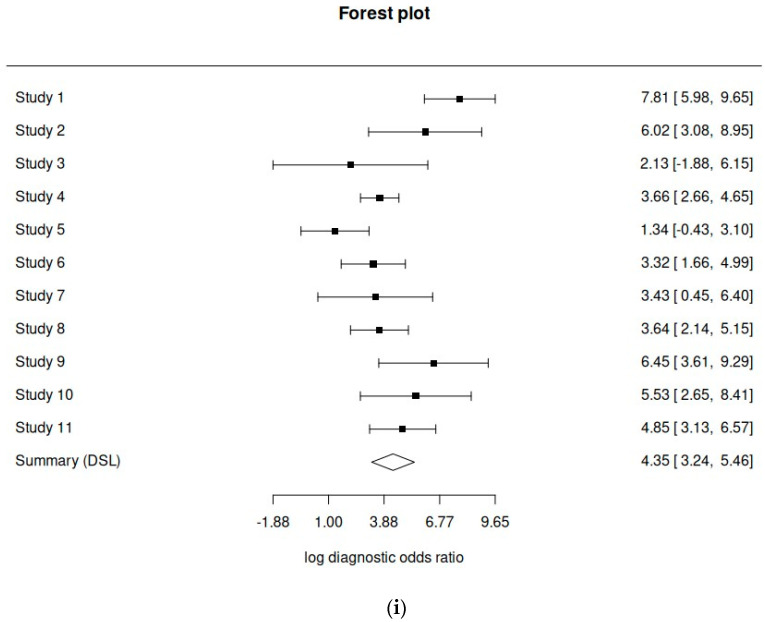

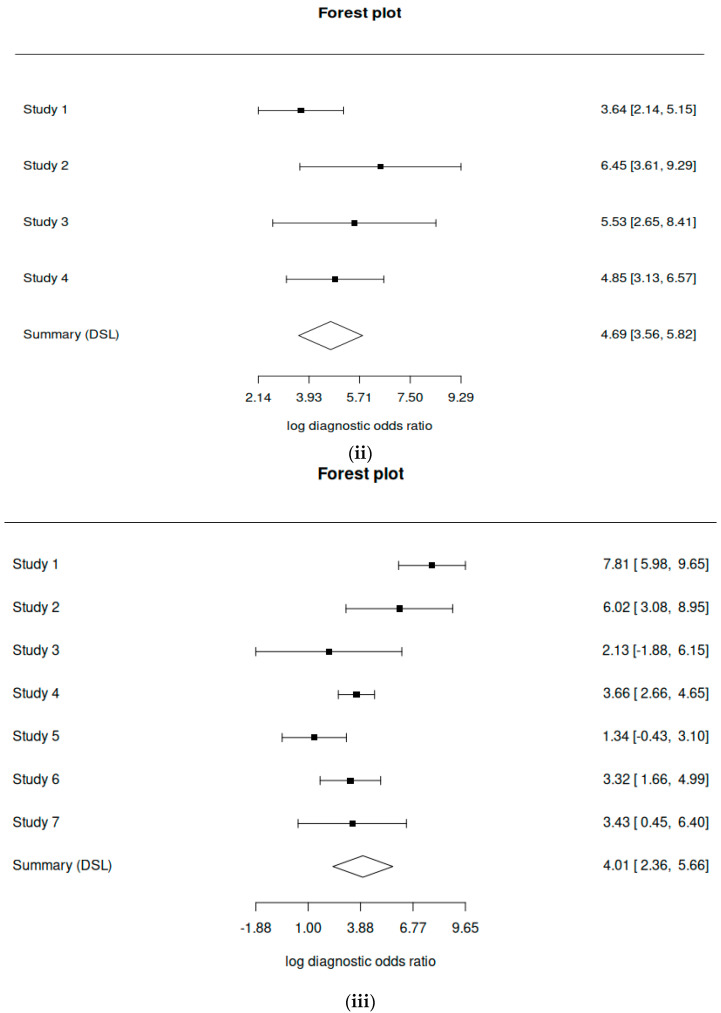
(**i**) Diagnostic accuracy ratio forest plot—all studies; (**ii**) diagnostic accuracy ratio forest plot—atypical hyperplasia; (**iii**) diagnostic accuracy ratio forest plot—endometrial cancer.

Among the included studies, 11 provided adequate data to be included in the statistical analysis. The pooled sensitivity was 0.863 (range 0.768–0.923), the pooled specificity was 0.916 (range 0.822–0.963), the overall DOR was 77.2 and the AUC was 0.948. Figure 2 shows the paired forest plot for the data. Figure 3 shows the confidence interval regions for the estimates of the primary studies, while Figure 4 presents the forest plot of the meta-analysis, using the DOR and the SROC curve. The diagnostic accuracy ratios for each of the included studies are presented in Figure 5.

Regarding heterogeneity, the results (Cochran’s Q: 10.488, 10 df, *p* = 0.399 and Higgins’ I^2^: 4.655%) revealed no significant heterogeneity, whereas the correlation analysis between sensitivity and false positive rate (rho: 0.306) indicated a low threshold effect possibility.

The subgroup analysis focused on endometrial cancer showed a pooled sensitivity of 0.886 (range 0.739 to 0.955) and a specificity of 0.862 with a range 0.055–0.695. The overall DOR was 54.9. The AUC was calculated to be 0.934. Cochran’s Q test was 6.212 (6 df, *p* = 0.4) and Higgin’s I^2^ was 3.42%. Moreover, the analysis for the diagnosis of atypical hyperplasia resulted in a pooled sensitivity of 0.816 (range 0.744–0.872) and a pooled specificity of 0.962 (range 0.987–0.895). The overall DOR was 109, the AUC was 0.939, Cochran’s Q test was 2.942 (3 df, *p* = 0.401) and Higgin’s I^2^ was 0%.

## 4. Discussion

According to the findings of the present study, frozen section shows high accuracy in the diagnosis of endometrial cancer. Additionally, with regard to atypical hyperplasia, our results revealed similar reliability for the examined diagnostic method. The finding of significantly high sensitivity and specificity in frozen section biopsy for the identification of both endometrial cancer and atypical hyperplasia not only underscores the validity of this diagnostic method but also highlights its clinical relevance. This enhanced sensitivity and specificity suggest that frozen section biopsy is a promising, reliable tool for accurately diagnosing hyperplasia or cancer within endometrial tissues.

Furthermore, the close approximation of the calculated area under the curve (AUC) values to 1 further reinforces the robustness and precision of frozen section biopsy. The AUC values, which serve as a quantitative measure of the diagnostic test’s discriminatory power, approach 1, which indicates an exceptionally high level of accuracy in distinguishing between affected and unaffected individuals.

These findings collectively emphasize the diagnostic superiority and clinical utility of frozen section biopsy in the context of endometrial cancer and atypical hyperplasia detection. As such, this method holds significant potential for informing clinical decision-making processes and facilitating timely and accurate interventions for patients with these conditions.

Moreover, intraoperative evaluation has been shown to be less time-consuming and is of low cost [59]. The necessity of intraoperative pathologist consultation (IC) is vital to assist in directing immediate surgical decisions. Such consultations offer surgeons vital insights that can potentially influence the course of a surgical procedure, including the possibility of modification or cessation. Frozen sections conducted during ICs serve various purposes such as defining the characteristics and scope of a lesion, assessing surgical margins, and ensuring that tissue sampling for subsequent investigations is adequate. Of note, other diagnostic methods should not be neglected, as they possess specific advantages. As an example, serum biomarkers (i.e., CA 125) have the capacity to identify endometrial cancer in patients experiencing abnormal uterine bleeding [60]. Nevertheless, the fact that frozen section is more accurate with a lower probability of yielding false-negative cases, an element that was also confirmed by the correlation test performed in our study, should be mentioned as one of its strongest points.

The early stages of endometrial cancer refer to those circumstances where cancer is confined within the uterus, not affecting adjacent tissues and organs. The standard clinical practice for treating stage I endometrial cancer is surgery, including the removal of the uterus, fallopian tubes and ovaries, as well as any nearby lymph nodes. According to the relevant literature, the five-year survival rate for stage IA is about 90%, with the prognosis being excellent and the cancer being highly curable with the surgical procedure alone [61]. Likewise, survival rates for IB stage demonstrate similar percentages [62]. Even though the prognosis of this stage remains high, the risk of a relapse is present, and in these cases, women may have to undergo additional treatment, such as radiation or chemotherapy.

An interesting point that strengthens the results of this meta-analysis is the design of the majority of the studies. In particular, all the included studies were observational; well-designed observational studies, whether retrospective or prospective, can provide valuable insights and evidence, especially in situations where randomized controlled trials are not practical or ethical. Furthermore, a strong feature of the present work is the identification of low heterogeneity between studies, a fact that facilitates and strengthens the generalizability of the results [63].

A strong feature of this meta-analysis is that, to our knowledge, no similar study has been carried out so far. On the other hand, this study bears certain limitations that need to be considered and addressed. The studies of this meta-analysis have included samples without calculating the required number of participants to obtain statistically significant results that can be generalized. In addition, the fact that different diagnostic techniques have been compared with endometrial frozen sections (e.g., transvaginal ultrasound, MRI) should not be neglected when synthesizing the findings.

## 5. Conclusions

The results of the present systematic review and meta-analysis showed that frozen section biopsy is a valid and reliable diagnostic tool for the diagnosis of endometrial cancer and atypical hyperplasia. In addition, it could provide information about tumor grade, which may have an important impact on clinical decision-making. Although other methods should not be overlooked, since they can be useful in clinical environments that do not have the possibility of applying other diagnostic methods, the revealing reliability of frozen section biopsy is unquestionable. In this regard, the clinical implementation of this method could enhance the quality of life and the level of care provided to women undergoing surgery.

## Figures and Tables

**Table 1:** Characteristics of the studies focusing on endometrial cancer that were included in this systematic review.

First Author, Year of Publication, Country	Journal	Study Design	Population of Cancer Patients	Preoperative Diagnosis	Sample Size	Control Group	Main Results
1. Boyraz et al., 2016, Turkey [13]	Balkan Med J	Retrospective cross-sectional	189 females with adenocarcinoma; 46 received a frozen section	Endometrial cancer	46	N/A	Frozen section’s sensitivity 54.4% (95% CI: 23.4–83.3), specificity 97.2% (95% CI: 85.1–99.9), positive predictive value was 85.7% (95% CI: 42.1–96.6) and negative predictive value was 87.5% (95% CI: 72.6–95.7).
2. Savelli et al., 2012, Italy [14]	Gynecologic Oncology	Retrospective cross-sectional	155 patients with endometrial cancer	Endometrial cancer	155	N/A	Sensitivity, accuracy, positive and negative predictive values were 92%, 92%, 89%, 94% and 92% for frozen section.
3. Fishman et al., 2000, USA [15]	European Journal of Gynaecological Oncology	Retrospective cross-sectional	91 grade I endometrial cancer- patients who received ultrasound and frozen section	Endometrial cancer, stage I	47	N/A	Myometrial invasion was accurately diagnosed in 41 out of 47 cases.
4. Desouki et al., 2017, USA [16]	American Journal for Clinical Pathology	Retrospective cross-sectional	205 patients, of whom 124 received frozen section during surgery and final biopsy.	Endometrial cancer	124	N/A	The agreement between frozen section and final biopsy in tumor grade was 80%. Predicting myometrial invasion was problematic with 36% underdiagnoses and 2.6% overdiagnoses by frozen sections.
5. Wang et al., 2016, USA [17]	International Journal of Gynecological Cancer	Retrospective cross-sectional	112 endometrial cancer patients who underwent hysterectomy and salpingo-oophorectomy	Endometrial cancer	112	N/A	Frozen section and final biopsy agreed 100%, 89.3%, 97.3% and 95.5%, respectively, for histological subtype, grade, myometrial invasion and tumor size.
6. Sala et al., 2014, Italy [18]	International Journal of Gynecological Cancer	Retrospect [19] ive cross-sectional	331 patients with grade I endometrial tumor who received frozen section during surgery	Endometrial cancer, stage I	331	N/A	Myometrial invasion detection was accurate in 93.9% of the cases for frozen section; 3.8% were underdiagnosed and 2.2% were overdiagnosed.
7. Gallego et al., 2014, Spain [20]	Abdominal Imaging	Retrospective cross-sectional	51 endometrial cancer patients who received MRI and frozen section during hysterectomy	Endometrial cancer	51	N/A	Sensitivity, specificity and accuracy of MRI for myometrial invasion were 90.2%, 77.8% and 97%, respectively, compared to 90.2%, 73.7% and 100% for frozen section.
8. Turan et al., 2013, Turkey [21]	European Journal of Obstetrics & Gynecology and Reproductive Biology	Retrospective cross-sectional	816 endometrial cancer patients	Endometrial cancer	816	N/A	Final biopsy was in agreement with frozen section in 89% of the cases.
9. Kumar et al., 2012, USA [22]	Gynecologic Oncology	Retrospective cross-sectional	784 endometrial cancer patients who underwent hysterectomy	Endometrial cancer	784	N/A	Final biopsy was in agreement with frozen section in 4% of the cases.
10. Ozturk et al., 2012, Turkey [23]	Archives of Gynecology and Obstetrics	Retrospective cross-sectional	220 endometrial cancer patients	Endometrial cancer	220	N/A	Sensitivity, specificity and accuracy were 73%, 96% and 90% for frozen section. Myometrial invasion sensitivity, specificity and accuracy of frozen section were 86%, 94% and 92%, respectively.
11. Kumar et al., 2011, USA [19]	PLoS One	Retrospective cross-sectional	146 endometrial cancer patients	Endometrial cancer	146	N/A	Frozen section results were accurate by 35% for tumor grade, 28% myometrial invasion, 13% for cervical involvement and 32% for lymphatic infiltration.
12. Papadia et al., 2009, USA [7]	International Journal of Gynecological Cancer	Retrospective cross-sectional	174 endometrial cancer patients	Endometrial cancer, stage I	174	N/A	Diagnostic accuracy of frozen section was 84.5% underdiagnoses and overdiagnoses were 9.5% and 6.0%, respectively.
13. Kucera et al., 2009, Czech Republic [24]	European Journal of Gynaecological Oncology	Retrospective cross-sectional	63 endometrial cancer patients who underwent hysterectomy and salpingo-oophorectomy	Endometrial cancer, stage I	63	N/A	Frozen section and final biopsy were in agreement for tumor grade in 85.7% of the cases.
14. Egle et al., 2008, Austria [25]	Gynecologic Oncology	Retrospective cross-sectional	318 endometrial cancer patients who received surgery	Endometrial cancer	303	N/A	Frozen section and final biopsy were in agreement in 95% of the cases.
15. Kir et al., 2004, Turkey [26]	European Journal of Gynaecological Oncology	Retrospective cross-sectional	55 patients with stage I endometrial cancer	Endometrial cancer, stage I	55	N/A	Frozen section and final biopsy were in agreement for 41 of the patients with respect to myometrial invasion and for 44 with respect to tumor grade.
16. Quinlivan et al., 2001, Australia [27]	BJOG: An International Journal of Obstetrics & Gynaecology	Retrospective cross-sectional	209 patients with endometrial cancer	Endometrial cancer	209	N/A	Tumor grade and myometrial invasion were accurate for 88.6% of cases analyzed by the frozen sections.
17. Kucera et al., 2000, Austria [28]	Gynecologic and Obstetric Investigation	Retrospective cross-sectional	70 endometrial cancer patients who received surgery	Endometrial cancer	70	N/A	Diagnostic accuracy and myometrial invasion were accurate for 80% and 84% of the cases.
18. Zorlu et al., 1993, Turkey [29]	Acta Obstetricia et Gynecologica Scandinavica	Retrospective cross-sectional	64 endometrial cancer patients	Endometrial cancer	64	N/A	Results of final biopsy for myometrial invasion were in agreement with 90.6% of the frozen sections’ results.
19. Doğan Durdağ et al., 2021, Turkey [30]	Archives of Gynecology and Obstetrics	Retrospective cross-sectional	223 endometrial cancer patients who received surgery	Endometrial cancer	223	N/A	Frozen sections’ accuracy was 76.23% for the subtype, 75.45% for the grade, 85.31% for myometrial invasion and 95.45% for tumor diameter.
20. Fotiou et al., 2009, Greece [31]	Gynecologic Oncology	Retrospective cross-sectional	142 stage I endometrial cancer patients who received surgery	Endometrial cancer, stage I	142	N/A	Myometrial invasion accuracy was 81.7%. False positives and false negatives were found at 17% and 21.9%, respectively.
21. Mao et al., 2008, China [32]	European Journal of Obstetrics & Gynecology and Reproductive Biology	Retrospective cross-sectional	424 endometrial cancer patients who received surgery	Endometrial cancer	424	N/A	Frozen section predicted the myometrial invasion with 90.3% accuracy.
22. Nakai et al., 2021, Japan [33]	BMC Cancer	Retrospective cross-sectional	231 endometrial cancer patients who received surgery	Endometrial cancer, stage III	172	N/A	Sensitivity, specificity and accuracy was 59.6%, 98.4% and 87.8% for preoperative sections compared to 55.3%, 99.2% and 87.2% of frozen sections.
23. Guo et al., 2022, China [34]	International Journal of Gynecology & Obstetrics	Retrospective cross-sectional	184 endometrial cancer patients who received surgery	Endometrial cancer	141	N/A	Frozen section was 87.23%, 81.15% and 98.2% accurate in the type, grade and myometrial invasion compared to final biopsy.
24. Giglio et al., 2020, USA [35]	Journal of the Society of Laparoscopic & Robotic Surgeons	Retrospective cross-sectional	105 cancer patients who received staging with robotic assistance	Endometrial cancer	75	N/A	Frozen sections were in agreement with the 80.6% of the final biopsies.
25. Bandala-Jacques et al., 2020, Mexico [36]	World Journal of Surgical Oncology	Retrospective cross-sectional	222 endometrial cancer patients who received surgery	Endometrial cancer, stage II	222	N/A	Frozen section was 76.13% accurate compared with final biopsy.
26. Iitsuka et al., 2021, Japan [37]	Journal of Obstetrics and Gynaecology Research	Retrospective cross-sectional	194 endometrial cancer patients who received surgery	Endometrial cancer	194	N/A	Frozen section was in agreement with MRI for 82% of the cases.
27. Gitas et al., 2019, Germany [38]	Archives of Gynecology and Obstetrics	Retrospective cross-sectional	164 endometrial cancer patients with stage I or II	Endometrial cancer, stages I or II	164	N/A	Cancer staging was accurate in 85.2% of cases for frozen sections compared to final biopsies, with 14% underdiagnoses and 0.8% overdiagnoses.
28. Abdallah et al., 2022, Libanon [39]	Journal of Obstetrics and Gynecology	Retrospective cross-sectional	245 patients who received hysterectomy for endometrial cancer	Endometrial cancer, stages I and II	70	Ν/A	Frozen and postoperative biopsy were in agreement by 92.3% for the subtype, 77% for tumor grade, 82% for myometrial invasion and 100% for tumor size.
29. Rei et al., 2020, Portugal [40]	Journal of Gynecology Obstetrics and Human Reproduction	Retrospective cross-sectional	187 endometrial cancer patients who received surgery	Endometrial cancer	156	N/A	Endo-vaginal ultrasound, MRI and frozen section had 56%, 71% and 67% sensitivity and 90%, 78% and 94% specificity. Frozen section was the method with the lowest percentage of underdiagnoses.
30. Sato et al., 2009, Japan [41]	The International Journal of Gynecological Cancer	Retrospective cross-sectional	191 endometrial cancer patients	Endometrial cancer	191	N/A	Frozen section was accurate for 162 patients; 8 were over-diagnosed and 21 underdiagnosed.
31. Ugaki et al., 2011, Japan [42]	International Journal of Gynecological Cancer	Retrospective cross-sectional	303 endometrial cancer patients who received surgery	Endometrial cancer	303	N/A	Accuracy for myometrial invasion was 77%. Diagnostic accuracy was 71%.
32. Çelik et al., 2010, Turkey [43]	International Journal of Gynecological Cancer	Retrospective cross-sectional	72 endometrial cancer patients	Endometrial cancer	72	N/A	Frozen section accuracy was 95.8% for the histological type and 90% for the grade.
33. Yanazume et al., 2011, Japan [44]	American Journal of Obstetrics & Gynecology	Retrospective cross-sectional	228 endometrial cancer patients who underwent hysterectomy	Endometrial cancer	228	N/A	Diagnostic accuracy of frozen section compared to final biopsy was 98% and 95% for the myometrial invasion.
34. Furukawa et al., 2010, Japan [45]	Archives of Gynecology and Obstetrics	Retrospective cross-sectional	168 endometrial cancer patients	Endometrial cancer	168	N/A	Diagnostic accuracy of frozen section was 85.7%; underdiagnoses and overdiagnoses were 9.5% and 4.8%, respectively.
35. Kanis et al., 2016, USA [46]	European Journal of Gynaecological Oncology	Retrospective cross-sectional	818 medical files of patients with endometrial cancer who received surgery	Endometrial cancer	285	N/A	Accuracy for myometrial invasion between frozen section and final biopsy was 95.5%.
36. Şenol et al., 2017, Turkey [47]	International Journal of Gynecological Pathology	Retrospective cross-sectional	150 patients with endometrial cancer who received diagnosis between 2010–2014	Endometrial cancer	150	N/A	Agreement between frozen section and final biopsy for 31 of the patients.
37. Karabagli et al., 2015, USA [48]	Archives of Gynecology and Obstetrics	Retrospective cross-sectional	79 endometrial carcinoma patients who received a frozen section during surgery	Endometrial cancer	79	N/A	Results of frozen section were in agreement in 89.9% for the grade, 88.6% for the myometrial invasion, 100% for cervical lesion, and 92.4% for lymphatic infiltration, compared to final biopsy.
38. Acikalin et al., 2015, Turkey [49]	Pathology & Oncology Research	Retrospective cross-sectional	291 patients who received frozen section and final biopsy for endometrial cancer	Endometrial cancer	291	N/A	Agreement between frozen section and final biopsy were 86%, 84.3% and 91.6% for histological subtype, tumor grade and myometrial invasion, respectively.
39. Kayıkçıoğlu et al., 2002, Turkey [50]	Acta Oncologica	Retrospective cross-sectional	154 patients with stage I endometrial cancer	Endometrial cancer, stage I	154	N/A	Accuracies for myometrial invasion and tumor grade were 87% and 85.7%, respectively.
40. Case et al., 2006, USA [51]	Obstetrics & Gynecology	Retrospective cross-sectional	36 patients with endometrial cancer or atypical hyperplasia	Endometrial cancer	36	N/A	Grade I lesions were upgraded in 61% of the cases for the frozen sections (95% CI 45–77). Tumor grade I cases were upgraded in 45% (98% CI 14–79) of the cases.
41. Stephan et al., 2014, USA [52]	Gynecologic Oncology	Retrospective cross-sectional	80 patients	Endometrial cancer	80	N/A	A total of 78/80 tumors remained endometrioid adenocarcinomas (97.5% correlation between FS and PS). When compared to PS, histological grade evaluation at the time of FS had 98% sensitivity and 53% specificity.

**Table 2:** Characteristics of the studies focusing on atypical hyperplasia that were included in this systematic review.

First Author, Year of Publication, Country	Journal	Study Design	Population of Cancer Patients	Preoperative Diagnosis	Sample Size	Control Group	Main Results
1. Indermaur et al., 2007, USA [53]	American Journal of Obstetrics & Gynecology	Retrospective cross-sectional	41 patients with atypical hyperplasia (surgery between 1987 and 2004).	Atypical hyperplasia	41	N/A	Final biopsy and frozen section were in agreement in 52.2% of the cases.
2. Morotti et al., 2012, Italy [54]	Gynecologic Oncology	Retrospective cross-sectional	Frozen section in 66 patients who underwent hysterectomy for atypical hyperplasia.	Atypical hyperplasia	66	N/A	Frozen section and hysterectomy were diagnosed tumors in 43.9% and 56% of the cases, respectively. A total of 94.1% of high-risk carcinomas were identified as endometrial cancer by the frozen section compared to 55% of low risk carcinomas.
3. Oz et al., 2014, Turkey [55]	The Asian Pacific Journal of Cancer Prevention	Retrospective cross-sectional	143 patients who underwent hysterectomy for atypical hyperplasia	Atypical hyperplasia	143	N/A	Frozen section and final biopsy were in agreement in 71% of the cases.
4. Gungorduk et al., 2015, Turkey [56]	Gynecologic and Obstetric Investigation	Retrospective cross-sectional	128 endometrial cancer patients who underwent hysterectomy due to atypical hyperplasia	Atypical hyperplasia	128	N/A	Diagnosis was accurate for 59 of the cases for the frozen section, whereas this was 69 for the final biopsy. The frozen section showed 29 patients with low and 30 with high tumor grade, while the final biopsy showed 38 and 30, respectively.
5. Kashyap et al., 2021, India [57]	European Journal of Obstetrics & Gynecology and Reproductive Biology	Case-control	80 patients who received hysterectomy for abnormal uterine bleeding	Atypical hyperplasia	40	40	Frozen section was accurate by 92.5% for detecting endometrial malignancies.
6. Turan et al., 2012, Turkey [58]	The Asian Pacific Journal of Cancer Prevention	Retrospective cross-sectional	125 patients who underwent surgery for atypical hyperplasia	Atypical hyperplasia	125	N/A	Final biopsy was in agreement with frozen section in 62.4% of the cases.
7. Stephan et al., 2014, USA [52]	Gynecologic Oncology	Retrospective cross-sectional	21 patients who underwent surgery for atypical hyperplasia	Atypical hyperplasia	21	N/A	Final biopsy was in agreement with frozen section in 55% of the cases (11/20).

## Data Availability

Data are available upon request.

## Supplementary Materials

The following supporting information can be downloaded at: https://www.mdpi.com/article/10.3390/cancers16061200/s1. Table S1: Quality evaluation of the included studies based on the QUADAS-2 tool.

## References

[1] Zhang S., Gong T.-T., Liu F.-H., Jiang Y.-T., Sun H., Ma X.-X., Zhao Y.-H., Wu Q.-J. (2019). Global, regional, and national burden of endometrial cancer, 1990–2017, results from the global burden of disease study, 2017. Front. Oncol..

[2] Olawaiye A.B., Cuello M.A., Rogers L.J. (2021). Cancer of the vulva, 2021 update. Int. J. Gynecol. Obstet..

[3] Nezhat C., Lavie O., Lemyre M., Gemer O., Bhagan L., Nezhat C. (2009). Laparoscopic hysterectomy with and without a robot, Stanford experience. JSLS J. Soc. Laparoendosc. Surg..

[4] Mariani A., Dowdy S.C., Cliby W.A., Gostout B.S., Jones M.B., Wilson T.O., Podratz K.C. (2008). Prospective assessment of lymphatic dissemination in endometrial cancer, a paradigm shift in surgical staging. Gynecol. Oncol..

[5] Seracchioli R., Solfrini S., Mabrouk M., Facchini C., Di Donato N., Manuzzi L., Savelli L., Venturoli S. (2010). Controversies in surgical staging of endometrial cancer. Obstet. Gynecol. Int..

[6] Cusimano M.C., Vicus D., Pulman K., Maganti M., Bernardini M.Q., Bouchard-Fortier G., Laframboise S., May T., Hogen L.F., Covens A.L. (2021). Assessment of sentinel lymph node biopsy vs lymphadenectomy for intermediate-and high-grade endometrial cancer staging. JAMA Surg..

[7] Papadia A., Azioni G., Brusacà B., Fulcheri E., Nishida K., Menoni S., Simpkins F., Lucci J.A., Ragni N. (2009). Frozen section underestimates the need for surgical staging in endometrial cancer patients. Int. J. Gynecol. Cancer..

[8] van de Poll-Franse L.V., Pijnenborg J.M.A., Boll D., Vos M.C., van den Berg H., Lybeert M.L.M., de Winter K., Kruitwagen R.F.P.M. (2012). Health related quality of life and symptoms after pelvic lymphadenectomy or radiotherapy vs. no adjuvant regional treatment in early-stage endometrial carcinoma, a large population-based study. Gynecol. Oncol..

[9] Page M.J., McKenzie J.E., Bossuyt P.M., Boutron I., Hoffmann T.C., Mulrow C.D., Shamseer L., Tetzlaff J.M., Akl E.A., Brennan S.E. (2021). The PRISMA 2020 statement, an updated guideline for reporting systematic reviews. Int. J. Surg..

[10] Whiting P.F., Rutjes A.W.S., Westwood M.E., Mallett S., Deeks J.J., Reitsma J.B., Leeflang M.M.G., Sterne J.A.C., Bossuyt P.M.M., QUADAS-2 Group (2011). QUADAS-2, a revised tool for the quality assessment of diagnostic accuracy studies. Ann. Intern. Med..

[11] Reitsma M.B., Flor L.S., Mullany E.C., Gupta V., Hay S.I., Gakidou E. (2021). Spatial, temporal, and demographic patterns in prevalence of smoking tobacco use and initiation among young people in 204 countries and territories, 1990–2019. Lancet Public Health.

[12] Doebler P., Holling H., Sousa-Pinto B. Meta-Analysis of Diagnostic Accuracy with Mada. In 2017, 30459830. https://cran.r-project.org/web/packages/mada/vignettes/mada.pdf.

[13] Boyraz G., Başaran D., Salman M.C., Özgül N., Yüce K. (2016). Does Preoperative Diagnosis of Endometrial Hyperplasia Necessitate Intraoperative Frozen Section Consultation?. Balkan Med. J..

[14] Savelli L., Testa A.C., Mabrouk M., Zannoni L., Ludovisi M., Seracchioli R., Scambia G., De Iaco P. (2012). A prospective blinded comparison of the accuracy of transvaginal sonography and frozen section in the assessment of myometrial invasion in endometrial cancer. Gynecol. Oncol..

[15] Fishman A., Altaras M., Bernheim J., Cohen I., Beyth Y., Tepper R. (2000). The value of transvaginal sonography in the preoperative assessment of myometrial invasion in high and low grade endometrial cancer and in comparison to frozen section in grade 1 disease. Eur. J. Gynaecol. Oncol..

[16] Desouki M.M., Li Z., Hameed O., Fadare O. (2017). Intraoperative Pathologic Consultation on Hysterectomy Specimens for Endometrial Cancer, An Assessment of the Accuracy of Frozen Sections, “Gross-Only” Evaluations, and Obtaining Random Sections of a Grossly “Normal” Endometrium. Am. J. Clin. Pathol..

[17] Wang X., Li L., Cragun J.M., Chambers S.K., Hatch K.D., Zheng W. (2016). Assessment of the Role of Intraoperative Frozen Section in Guiding Surgical Staging for Endometrial Cancer. Int. J. Gynecol. Cancer.

[18] Sala P., Morotti M., Menada M.V., Cannavino E., Maffeo I., Abete L., Fulcheri E., Menoni S., Venturini P., Papadia A. (2014). Intraoperative frozen section risk assessment accurately tailors the surgical staging in patients affected by early-stage endometrial cancer, The application of 2 different risk algorithms. Int. J. Gynecol. Cancer.

[19] Kumar S., Bandyopadhyay S., Semaan A., Shah J.P., Mahdi H., Morris R., Munkarah A., Ali-Fehmi R. (2011). The role of frozen section in surgical staging of low risk endometrial cancer. PLoS ONE.

[20] Gallego J.C., Porta A., Pardo M.C., Fernández C. (2014). Evaluation of myometrial invasion in endometrial cancer, comparison of diffusion-weighted magnetic resonance and intraoperative frozen sections. Abdom. Imaging.

[21] Turan T., Oguz E., Unlubilgin E., Tulunay G., Boran N., Demir O.F., Kose M.F. (2013). Accuracy of frozen-section examination for myometrial invasion and grade in endometrial cancer. Eur. J. Obstet. Gynecol. Reprod. Biol..

[22] Kumar S., Medeiros F., Dowdy S.C., Keeney G.L., Bakkum-Gamez J.N., Podratz K.C., Cliby W.A., Mariani A. (2012). A prospective assessment of the reliability of frozen section to direct intraoperative decision making in endometrial cancer. Gynecol. Oncol..

[23] Ozturk E., Dikensoy E., Balat O., Ugur M.G., Aydin A. (2012). Intraoperative frozen section is essential for assessment of myometrial invasion but not for histologic grade confirmation in endometrial cancer, a ten-year experience. Arch. Gynecol. Obstet..

[24] Kucera E., Václav H., Radovan T., Otcenásek M., Drahonovský J., Feyereisl J. (2009). Accuracy of intraoperative frozen section during laparoscopic management of early endometrial cancer. Eur. J. Gynaecol. Oncol..

[25] Egle D., Grissemann B., Zeimet A.G., Müller-Holzner E., Marth C. (2008). Validation of intraoperative risk assessment on frozen section for surgical management of endometrial carcinoma. Gynecol. Oncol..

[26] Kir G., Kir M., Cetiner H., Karateke A., Gurbuz A. (2004). Diagnostic problems on frozen section examination of myometrial invasion in patients with endometrial carcinoma with special emphasis on the pitfalls of deep adenomyosis with carcinomatous involvement. Eur. J. Gynaecol. Oncol..

[27] Quinlivan J.A., Petersen R.W., Nicklin J.L. (2001). Accuracy of frozen section for the operative management of endometrial cancer. Br. J. Obstet. Gynaecol..

[28] Kucera E., Kainz C., Reinthaller A., Sliutz G., Leodolter S., Kucera H., Breitenecker G. (2000). Accuracy of intraoperative frozen-section diagnosis in stage I endometrial adenocarcinoma. Gynecol. Obstet. Investig..

[29] Zorlu C.G., Kuscu E., Ergun Y., Aydogdu T., Cobanoglu O., Erdas O. (1993). Intraoperative evaluation of prognostic factors in stage I endometrial cancer by frozen section, how reliable?. Acta Obstet. Gynecol. Scand..

[30] Doğan Durdağ G., Alemdaroğlu S., Aka Bolat F., Yılmaz Baran Ş., Yüksel Şimşek S., Çelik H. (2021). Accuracy of intra-operative frozen section in guiding surgical staging of endometrial cancer. Arch. Gynecol. Obstet..

[31] Fotiou S., Vlahos N., Kondi-Pafiti A., Zarganis P., Papakonstantinou K., Creatsas G. (2009). Intraoperative gross assessment of myometrial invasion and cervical involvement in endometrial cancer, Role of tumor grade and size. Gynecol. Oncol..

[32] Mao Y., Wan X., Chen Y., Lv W., Xie X. (2008). Evaluation of the accuracy of intra-operative gross examination for the surgical management of endometrial cancer. Eur. J. Obstet. Gynecol. Reprod. Biol..

[33] Nakai G., Tanaka Y., Yamada T., Ohmichi M., Yamamoto K., Osuga K. (2021). Can addition of frozen section analysis to preoperative endometrial biopsy and MRI improve identification of high-risk endometrial cancer patients?. BMC Cancer.

[34] Guo Q., Yi H., Chen X., Song J., Chen L., Zheng X. (2022). Is routine frozen section analysis necessary in patients with non-endometrioid cancer or grade 3 endometrioid cancer?. Int. J. Gynecol. Obstet..

[35] Giglio A., Miller B., Curcio E., Kuo Y.H., Erler B., Bosscher J., Hicks V., ElSahwi K. (2020). Challenges to intraoperative evaluation of endometrial cancer. J. Soc. Laparosc. Robot. Surg..

[36] Bandala-Jacques A., Cantú-de-León D., Pérez-Montiel D., Salcedo-Hernández R.A., Prada D., González-Enciso A., Gonzalez-Valdés A., Barquet-Muñoz S.A. (2020). Diagnostic performance of intraoperative assessment in grade 2 endometrioid endometrial carcinoma. World J. Surg. Oncol..

[37] Iitsuka C., Asami Y., Hirose Y., Nagashima M., Mimura T., Miyamoto S., Onuki M., Ohgiya Y., Kushima M., Sekizawa A. (2021). Preoperative Magnetic Resonance Imaging versus Intraoperative Frozen Section Diagnosis for Predicting the Deep Myometrial Invasion in Endometrial Cancer, Our Experience and Literature Review. J. Obstet. Gynaecol. Res..

[38] Gitas G., Proppe L., Alkatout I., Rody A., Kotanidis C., Tsolakidis D., Baum S. (2019). Accuracy of frozen section at early clinical stage of endometrioid endometrial cancer, a retrospective analysis in Germany. Arch. Gynecol. Obstet..

[39] Abdallah R., Khalil A., Ghunaim S., El Housheimi A., Khalife D., Sassine D., Khoury K., Mailhac A., Nassour F., Saliba M. (2022). The accuracy and clinical impact of intraoperative frozen section in determining the extent of surgical intervention in patients with early stage endometrial cancer. J. Obstet. Gynaecol..

[40] Rei M., Rodrigues I., Condeço P., Igreja F., Veríssimo C., Mendinhos G. (2020). Endometrial cancer, Preoperative versus intraoperative staging. J. Gynecol. Obstet. Hum. Reprod..

[41] Sato S., Itamochi H., Shimada M., Fujii S., Naniwa J., Uegaki K., Sato S., Nonaka M., Ogawa T., Kigawa J. (2009). Preoperative and intraoperative assessments of depth of myometrial invasion in endometrial cancer. Int. J. Gynecol. Cancer.

[42] Ugaki H., Kimura T., Miyatake T., Ueda Y., Yoshino K., Matsuzaki S., Fujita M., Kimura T., Morii E., Enomoto T. (2011). Intraoperative frozen section assessment of myometrial invasion and histology of endometrial cancer using the revised FIGO staging system. Int. J. Gynecol. Cancer.

[43] Celik C., Ozdemir S., Esen H., Balci O., Ylmaz O. (2010). The clinical value of preoperative and intraoperative assessments in the management of endometrial cancer. Int. J. Gynecol. Cancer.

[44] Yanazume S., Saito T., Eto T., Yamanaka T., Nishiyama K., Okadome M., Ariyosh K. (2011). Reassessment of the utility of frozen sections in endometrial cancer surgery using tumor diameter as an additional factor. Am. J. Obstet. Gynecol..

[45] Furukawa N., Takekuma M., Takahashi N., Hirashima Y. (2010). Intraoperative evaluation of myometrial invasion and histological type and grade in endometrial cancer, diagnostic value of frozen section. Arch. Gynecol. Obstet..

[46] Kanis M.J., Rahaman J., Moshier E.L., Zakashansky K., Chuang L., Kolev V. (2016). Detection and correlation of pre-operative, frozen section, and final pathology in high-risk endometrial cancer. Eur. J. Gynaecol. Oncol..

[47] Şenol T., Polat M., Özkaya E., Karateke A. (2017). Misinterpretation of Frozen Section in Endometrial Cancer Cases, Does It Have Any Effect on Disease-free and Overall Survival?. Int. J. Gynecol. Pathol..

[48] Karabagli P., Ugras S., Yilmaz B.S., Celik C. (2015). The evaluation of reliability and contribution of frozen section pathology to staging endometrioid adenocarcinomas. Arch. Gynecol. Obstet..

[49] Acikalin A., Gumurdulu D., Bagir E.K., Torun G., Guzel A.B., Zeren H., Vardar M.A. (2015). The Guidance of Intraoperative Frozen Section For Staging Surgery in Endometrial Carcinoma, Frozen section in endometrial carcinoma. Pathol. Oncol. Res..

[50] Kayikçioǧlu F., Boran N., Meydanli M.M., Tulunay G., Köse F.M., Bülbül D. (2002). Is frozen-section diagnosis a reliable guide in surgical treatment of stage I endometrial carcinoma?. Acta Oncol..

[51] Case A., Rocconi R., Straughn M.J., Conner M., Novak L., Wang W., Huh W. (2006). A prospective blinded evaluation of the accuracy of frozen section for the surgical management of endometrial cancer. Obstet. Gynecol..

[52] Stephan J.M., Hansen J., Samuelson M., McDonald M., Chin Y., Bender D., Reyes H.D., Button A., Goodheart M.J. (2014). Intra-operative frozen section results reliably predict final pathology in endometrial cancer. Gynecol. Oncol..

[53] Indermaur M.D., Shoup B., Tebes S., Lancaster J.M. (2007). The accuracy of frozen pathology at time of hysterectomy in patients with complex atypical hyperplasia on preoperative biopsy. Am. J. Obstet. Gynecol..

[54] Morotti M., Menada M.V., Moioli M., Sala P., Maffeo I., Abete L., Fulcheri E., Menoni S., Venturini P., Papadia A. (2012). Frozen section pathology at time of hysterectomy accurately predicts endometrial cancer in patients with preoperative diagnosis of atypical endometrial hyperplasia. Gynecol. Oncol..

[55] Oz M., Ozgu E., Korkmaz E., Bayramoglu H., Erkaya S., Gungor T. (2014). Utility of frozen section pathology with endometrial pre-malignant lesions. Asian Pac. J. Cancer Prev..

[56] Gungorduk K., Ozdemir A., Ertas I., Şahbaz A., Aşıcıoğlu O., Gokcu M., Solmaz U., Harma M., Uzunçakmak C., Dogan A. (2015). A novel preoperative scoring system for predicting endometrial cancer in patients with complex atypical endometrial hyperplasia and accuracy of frozen section pathological examination in this context, A multicenter study. Gynecol. Obstet. Investig..

[57] Kashyap A., Rajaram S., Gupta B., Arora V.K., Upreti L., Jain S. (2021). Evaluation of frozen section biopsy for fast track diagnosis of endometrial pathology in high-risk women with abnormal uterine bleeding. Eur. J. Obstet. Gynecol. Reprod. Biol..

[58] Turan T., Karadag B., Karabuk E., Tulunay G., Ozgul N., Gultekin M., Boran N., Isikdogan Z., Kose M.F. (2012). Accuracy of frozen sections for intraoperative diagnosis of complex atypical endometrial hyperplasia. Asian Pac. J. Cancer Prev..

[59] Mahe E., Ara S., Bishara M., Kurian A., Tauqir S., Ursani N., Vasudev P., Aziz T., Ross C., Lytwyn A. (2013). Intraoperative pathology consultation, error, cause and impact. Can. J. Surg..

[60] Nithin K.U., Sridhar M.G., Srilatha K., Habebullah S. (2018). CA 125 is a better marker to differentiate endometrial cancer and abnormal uterine bleeding. Afr. Health Sci..

[61] Lajer H., Elnegaard S., Christensen R.D., Ortoft G., Schledermann D.E., Mogensen O. (2012). Survival after stage IA endometrial cancer, can follow-up be altered? A prospective nationwide Danish survey. Acta Obstet. Gynecol. Scand..

[62] Rahatli S., Dizdar O., Kucukoztas N., Oguz A., Yalcin S., Ozen O., Reyhan N.H., Tarhan C., Yildiz F., Dursun P. (2014). Good outcomes of patients with stage IB endometrial cancer with surgery alone. Asian Pac. J. Cancer Prev..

[63] Fletcher J. (2007). What is heterogeneity and is it important?. BMJ.

