# Supplementary material for: Accuracy of Frozen Section Biopsy in the Diagnosis of Endometrial Cancer: A Systematic Review and Meta-Analysis
Source: Cancers (Basel). 2024 Mar 19;16(6):1200. doi: 10.3390/cancers16061200 (PMC10969579; doi:10.3390/cancers16061200)
Supplement: Supplementary file 1 [file cancers-16-01200-s001.zip › cancers-2891037-supplementary.pdf]

Supplementary Table 1. Quality evaluation of the included studies based on the QUADAS-2 tool.

Study	Risk of bias				Application concerns		
	Patient selection	Index test	Reference standard	Flow & timing	Patient selection	Index test	Reference standard
1.Boyraz, 2016	High	Low	Low	Low	Low	Low	Low
2.Fishman, 2000	High	Low	Low	Low	Low	Low	Low
3.Desouki, 2017	High	Low	Low	Low	Low	Low	Low
4.Şenol, 2017	High	Low	Low	Low	Low	Low	Low
5.Kanis, 2016	High	Low	Low	Low	Low	Low	Low
6.Wang, 2016	High	Low	Low	Low	Low	Low	Low
7.Karabagli, 2015	High	Low	Low	Low	Low	Low	Low
8.Sala, 2014	High	Low	Low	Low	Low	Low	Low
9.Acikalin, 2015	High	Low	Low	Low	Low	Low	Low
10.Gallego, 2014	High	Low	Low	Low	Low	Low	Low
11.Turan, 2013	High	Low	Low	Low	Low	Low	Low
12.Kumar, 2012	High	Low	Low	Low	Low	Low	Low
13.Ozturk, 2012	High	Low	Low	Low	Low	Low	Low
14.Kumar, 2011	High	Low	Low	Low	Low	Low	Low
15.Yanazume, 2011	High	Low	Low	Low	Low	Low	Low
16.Celik, 2010	High	Low	Low	Low	Low	Low	Low
17.Papadia, 2009	High	Low	Low	Low	Low	Low	Low
18.Furukawa, 2010	High	Low	Low	Low	Low	Low	Low
19.Kucera, 2009	High	Low	Low	Low	Low	Low	Low
20.Egle, 2008	High	Low	Low	Low	Low	Low	Low
21.Kir, 2004	High	Low	Low	Low	Low	Low	Low
22.Kayıkçıoğlu, 2002	High	Low	Low	Low	Low	Low	Low
23.Quinlivan, 2001	High	Low	Low	Low	Low	Low	Low
24.Kucera, 2000	High	Low	Low	Low	Low	Low	Low
25.Zorlu, 1993	High	Low	Low	Low	Low	Low	Low
26.Durdağ, 2021	High	Low	Low	Low	Low	Low	Low
27.Ugaki, 2011	High	Low	Low	Low	Low	Low	Low
28.Fotiou, 2009	High	Low	Low	Low	Low	Low	Low
29.Sat, 2009	High	Low	Low	Low	Low	Low	Low
30.Mao, 2008	High	Low	Low	Low	Low	Low	Low
31.Nakai, 2021	High	Low	Low	Low	Low	Low	Low
32.Iitsuka, 2021	High	Low	Low	Low	Low	Low	Low
33.Guo, 2022	High	Low	Low	Low	Low	Low	Low
34.Bandala-Jacques, 2020	High	Low	Low	Low	Low	Low	Low
35.Giglio, 2020	High	Low	Low	Low	Low	Low	Low
36.Rei, 2019	High	Low	Low	Low	Low	Low	Low
37.Santoro, 2019	High	Low	Low	Low	Low	Low	Low
38.Gitas, 2019	High	Low	Low	Low	Low	Low	Low
39.Kashyap, 2021	High	Low	Low	Low	Low	Low	Low
40.Abdallah, 2022	High	Low	Low	Low	Low	Low	Low
41.Case, 2014	High	Low	Low	Low	Low	Low	Low

42.Indermaur, 2007	High	Low	Low	Low	Low	Low	Low
44.Oz, 2014	High	Low	Low	Low	Low	Low	Low
45.Gungorduk, 2015	High	Low	Low	Low	Low	Low	Low
46.Kashyap, 2021	High	Low	Low	Low	Low	Low	Low
47.Turan, 2012	High	Low	Low	Low	Low	Low	Low
48.Stephan, 2014	High	Low	Low	Low	Low	Low	Low
49. Morotti, 2012	High	Low	Low	Low	Low	Low	Low
